# Alveolar rhabdomyosarcoma of epididymis: A case report

**DOI:** 10.3389/fonc.2022.1027504

**Published:** 2022-11-02

**Authors:** Xiangyi Liang, Shun Wang, Tao Li, Longlong Liu, Yu Duan, Yuting Luo, Qing Wang, Jianxin Hu, Kehua Jiang

**Affiliations:** Guizhou Provincial People’s Hospital, Guizhou, China

**Keywords:** alveolar, rhabdomyosarcoma, epididymis, diagnosis, treatment

## Abstract

Rhabdomyosarcoma (RMS) is a soft tissue tumor, which is the most common in the head, neck, limbs, and trunk. RMS originating from the epididymis is extremely rare. Herein, we reported a 34-year-old patient with RMS on the right epididymis. For this case, right epididymal mass resection was performed and intraoperative freezing suggested a malignant tumor. Right testicular radical resection was subsequently adopted, with right epididymal alveolar RMS being pathologically diagnosed. Alternating VAC/VI chemotherapy was given after surgery, and tumor recurrence has not been found so far.

## Introduction

Rhabdomyosarcoma (RMS) is an extremely rare solid malignant tumor, it tends to metastasize early and is difficult to differentiate from other epididymal tumors. In general, RMS accounts for 5%-10% of childhood tumors, while only 7% originate from epididymis (1).Epididymal RMS (eRMS) often causes unilateral inguinal canal or scrotal swelling. We herein reported a patient with right alveolar eRMS which presented right scrotal swelling as the first symptom.

## Case report

**Figure 1:**
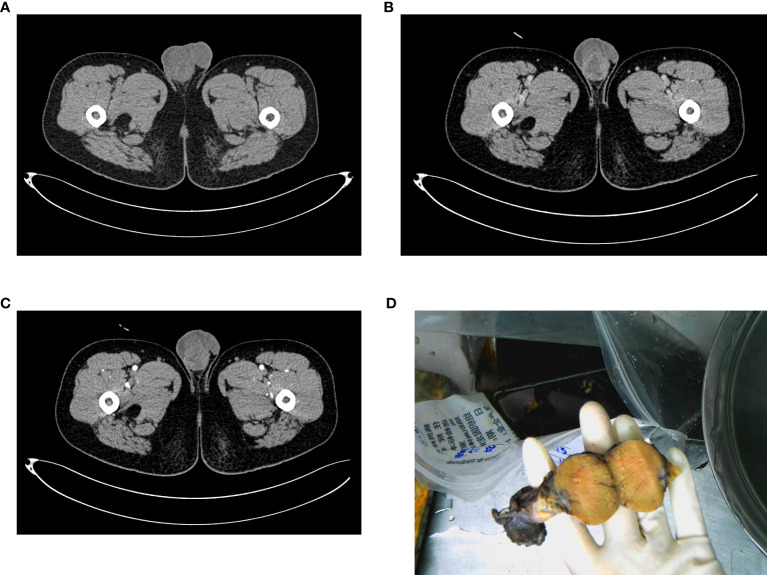
Imaging presentation and gross specimen of a patient with primary eRMS. **(A)** CT plain scan showed the enlarged and inhomogeneous density of the right testicle; **(B)** (arterial phase) and **(C)** (venous phase): CT enhanced scan showed inhomogeneous enhancement of the occupying lesion; **(D)** General observation.

**Figure 2:**
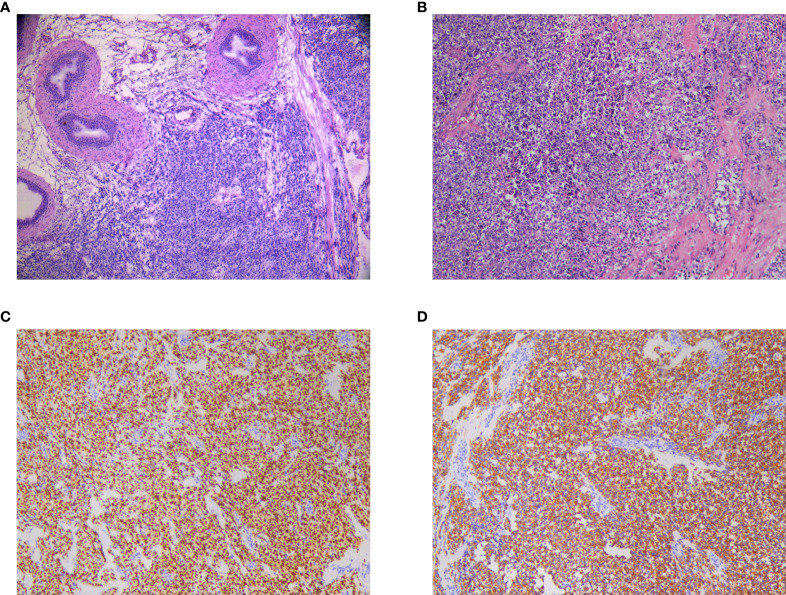
Microscopic pathology of primary eRMS. **(A)** Heterotypic cells were seen between the tissues of the right epididymis; **(B)** tumor size was about 3.5×3.2×2.5 cm; **(C)** Desmin (+); **(D)** Myogenin (+).

A 34-year-old male was admitted to our department for an enlarged right scrotum with a feeling of swelling. Physical examination revealed that the right scrotum was slightly enlarged, with a hard mass (about 4.0×3.0 cm) touched in the right epididymis. The right scrotum was palpated with slight pain, its mobility was poor, but the surface was smooth. An enhanced CT scan observed a tumor in the dorsal lower part of the testicle (3.3×3.0 cm), with heterogeneous enhancement found (**Figure 1**). Enlarged lymph nodes were not found in the imaging examination. The tumor markers α-fetoprotein (AFP), human chorionic gonadotropin (HCG), and serum lactate dehydrogenase (LDH) were within normal values. Right epididymal mass was primarily resected and a malignant tumor was considered by intraoperative freezing, while a radical operation was then adapted. The pathological results showed that a gray solid tumor was seen in the right epididymal section (**Figure 1D**), and tumor cells infiltrated among epididymis (**Figure 2A**), the tumor was completely removed and the margin of vas deferens and spermatic cord were negative. The final immunohistochemical staining was as follows: Vimentin, Desmin Myogenin, MyoD1, SMA, CD56, ALK, S-100 polyclonal (+), Fli-1 (weak +), CD99 (partial cell +), Ki-67 (40%-60% +), WT-1 (-), CK wide (-), EMA (-) (**Figure 2**); FOX gene translocation (+). The patient was diagnosed as alveolar eRMS. VAC (vincristine 2 mg d1、d8、d15+ adriamycin 3 mg d1+ cyclophosphamide 2.1 g d1)/VI (vincristine 2 mg d1、d8、d15+ irinotecan 80 mg d1-d5) alternating chemotherapy was performed every three weeks. Regularly followed was performed after six cycles of VAC/VI chemotherapy and signs of tumor recurrence or progress were not observed.

## Discussion

Scrotal RMS primarily originates from particular tissues in children and adolescents. Early diagnosis is critical given its aggressive nature and the relationship between tumor staging and survival. Unilateral scrotal painless swelling is the typical symptom like this patient, while the swelling pain always comes from accompanied epididymitis (1). Ultrasound can accurately identify testicular and epididymal tumors, but can not clarify the tumors’ nature. CT or Magnetic resonance imaging (MRI) is primarily recommended, however, none of them can distinguish RMS from other testicular carcinomas. Moreover, there is no specific serum tumor marker for epididymal RMS at present, thus the HCG and AFP are both negative which only help to eliminate the germ cell tumors. Therefore, RMS was only depended on the finally pathological diagnosis (2), which is characterized by one or more positive muscle-specific markers like desmin, muscle-specific actin, myoglobin or myogenin (3, 4).

RMS is a mesenchymal carcinoma which arises from skeletal muscle cells (5). The most common RMS types is embryonal, which involves varying degrees of embryonic rhabdogenesis shows different “loose and dense” cells in the myxoid matrix. Others RMS types include alveolar, botryoid embryonal, spindle cell embryonal, and anaplastic (6). The botryoid RMS forms grape-like polypoid masses lined by epithelium and reveals heightened subepithelial cellularity. Anaplastic type contains bundles of large and variably myogenic spindle cells with hyperchromatic and irregular nuclei and prominent nucleoli. For alveolar RMS, it demonstrates a vague resemblance to fetal alveoli as a result of intersecting fibrous septa with interseptal nesting and discohesion (7). This patient was diagnosed as alveolar eRMS combined with imaging and postoperative pathological findings. Current studies also reported that 20% RMS arised from genitourinary system, especially from epididymis or spermatic cord among adult (5).

The eRMS management is multimodal. Complete surgical resection followed by adjuvant chemotherapy is currently considered to be more appropriate (8). Recommendation for localized eRMS is based on clinical trials conducted by cooperative groups in North America and Europe, that the surgical approach is uniformed to withdrawal the primary tumor from inguinal orchiectomy with high semen cord ligation (9). Unilateral radical scrotal excision with scrotal skin is recommended if the scrotum is invaded (8, 10). A total of 255 eRMS cases were reported by the National Cancer Institute’s Surveillance, Epidemiology, and End Results (SEER) program from 1973 to 2009. For patients aged ≥10 years, lymph nodes dissection was found to improve five-year survival rate (OS) from 64%-86% (P < 0.01). On the contrary, as patients aged< 10 years have better prognosis, the five-year OS for lymph node dissection or not was 100% and 97%, respectively (P = 0.37) (11). Thus retroperitoneal lymph node dissection (RPLND) is recommended for patients aged≥ 10 years, but not suggest if enlarged lymph nodes were not demonstrated by CT scan when patients <10 years. Even though, the consensus on eRMS management is controversial. For this patient, RPLND was not performed for this patient as the limited evidence and absence of enlarged lymph node in preoperative CT scan.

RMS is a chemotherapy-sensitive tumor with VAC regimens (vincristine+adriamycin+cyclophosphamide) being the most common regimen to improve survival and reduce recurrence (12, 13). For intermediate-risk cases, VAC/VI (vincristine+irinotecan) alternation does not improve survival but reduces cumulative dose of cyclophosphamide and decreases hematologic toxicity (14). According to the tumor-nodes metastases classification and Intergroup Rhabdomyosarcoma Study system, this patient was classified as stage I b (intermediate-risk group) and alternating VAC/VI chemotherapy was selected (15). Adjuvant radiotherapy is also effective for residual lesions, lymph node metastases, local recurrence, and distant metastatic lesions. However, it only improves survival for patients with lymph node involvement (12), thus, adjuvant radiotherapy is not applied.

The main factors affecting the prognosis of eRMS are age, tumor size, pathological type, local recurrence, metastasis, and chemotherapy (16, 17). Its 5-year survival rate is 22.2% for those with recurrence/metastasis, and 94.6% for those without (18). The prognosis in adult is worse than children, with a 5-year tumor-free survival and 5-year OS rates being found 28% and 40%, respectively (19). Moreover, the embryonal has a better prognosis, which followed by pleomorphic and spindle cell types, while the glandular follicular type was the worst (18).

In summary, eRMS is rare and rapidly progressing. Its clinical symptom and imaging manifestation are not specific. So, it is often confused with epididymitis, epididymal tuberculosis, and epididymal tumor, which finally leads misdiagnosis and delays treatment. Patients with scrotal painless swelling, testicular hydrocele, or rapidly growing scrotal mass who is poorly respond to antibiotic should be considered with eRMS. Radical orchiectomy and adjuvant chemotherapy can reduce recurrence and improve survival. Finally, the patient should be monitored for a long time considering RMS is easy to metastasise.

This study was funded by National Natural Science Foundation of China (Number: 82060462), Science and Technology Plan project of Guizhou Province (Number: [2019]5405), and the Doctoral Foundation of Guizhou Provincial People’s Hospital (GZSYBS[2018]02). The funding agencies and donors had no role in any aspect of this study.

## Data availability statement

The raw data supporting the conclusions of this article will be made available by the authors, without undue reservation.

## Author contributions

XL, SW and LL obtained and analyzed the clinical data. XL, SW and TL wrote the manuscript. KJ and JH designed and constructed the figures. KJ, QW designed the study, study supervision and edited the manuscript. All authors contributed to writing and revising the manuscript and figures. All authors contributed to the article and approved the submitted version.

## Funding

This study was also funded by Science and Technology Support Plan of Guizhou Province in 2020 (Fund No. : Guizhou Science and Technology Support [2020]4Y142).

## Conflict of interest

The authors declare that the research was conducted in the absence of any commercial or financial relationships that could be construed as a potential conflict of interest.

## Publisher’s note

All claims expressed in this article are solely those of the authors and do not necessarily represent those of their affiliated organizations, or those of the publisher, the editors and the reviewers. Any product that may be evaluated in this article, or claim that may be made by its manufacturer, is not guaranteed or endorsed by the publisher.
